# Exploring Home-Visit Rehabilitation for Terminally Ill Patients With Cancer: Insights From Japanese Care Managers

**DOI:** 10.7759/cureus.76978

**Published:** 2025-01-05

**Authors:** Satoko Sakaguchi, Fumiko Kaneko, Kazuya Saita, Hitoshi Okamura

**Affiliations:** 1 Department of Psychosocial Rehabilitation, Graduate School of Biomedical and Health Sciences, Hiroshima University, Hiroshima, JPN; 2 Department of Psychiatry, Graduate School of Biomedical and Health Sciences, Hiroshima University, Hiroshima, JPN

**Keywords:** cancer, care manager, home-visit rehabilitation, palliative rehabilitation, terminally ill

## Abstract

Background: Care managers (CMs) play a vital role in planning, managing, providing family support, and coordinating services for individuals in need, particularly in the field of palliative care. To implement home-visit rehabilitation for terminally ill patients with cancer effectively, it is essential for the care plan created by the CM to be incorporated into the system. Collaboration with the CM is key to ensuring the successful implementation of rehabilitation. Despite this necessity, there is a lack of studies investigating the background and challenges preventing progress in this area. Therefore, this study aimed to clarify the current status of home-visit rehabilitation for terminally ill patients with cancer, focusing on CMs. By identifying associated factors, we aim to suggest guidelines for promoting the use of home-visit rehabilitation to maintain and improve the quality of life of terminally ill patients with cancer in their home settings.

Methods: A self-administered survey was mailed or distributed directly to CMs. It collected the following 11 types of information: gender, age, years of work experience, basic job title, working form, office management system, experience with home-visit rehabilitation (including for terminally ill patients with cancer), perception and importance of this service, and reasons for not implementing it for terminally ill patients with cancer. T-tests or chi-square tests were conducted to examine the association between home-visit rehabilitation experiences and sociodemographic data, as well as the relationship between home-visit rehabilitation for terminally ill patients with cancer, sociodemographic data, and perceptions of and need for home-visit rehabilitation.

Results: Out of 520 questionnaires distributed across seven regions in three prefectures, 388 were returned, resulting in a response rate of 74.6%. After excluding incomplete responses, data from 357 respondents were considered valid. Age (p = 0.004), years of work experience (p < 0.001), and working form (p < 0.001) were found to be related to home-visit rehabilitation experience. A significant association was identified between home-visit rehabilitation experience for terminally ill patients with cancer and years of work experience (p < 0.001), and the perception (p < 0.001) and necessity (p = 0.001) for home-visit rehabilitation.

Conclusions: These results suggest that promoting palliative rehabilitation for terminally ill patients with cancer in home care requires actively encouraging CMs to develop a shared understanding of the services and their role.

## Introduction

The population of Japan is expected to decline after peaking in 2008, but aging will continue to rise rapidly. The aging rate is projected to increase from 20.2% in 2020 to 30.3% in 2025 and 35.3% in 2040, surpassing that of major European countries and the United States [1,2]. This demographic shift presents a significant challenge, particularly regarding the increasing number of deaths among older adults and the urgent need for adequate support.

The Ministry of Health, Labor and Welfare's Survey on Attitudes Toward Medical Care in the Final Stage of Life [3] revealed a strong desire among many individuals to remain at home even when requiring nursing care. Despite this preference, a significant number of people ultimately die in hospitals [1]. This trend is not unique to Japan but is also observed in other countries [4, 5]. Providing medical care services and creating a conducive environment for individuals to remain at home, whether in Japan or elsewhere, is crucial.

In Japan, individuals in need of care can receive a range of services, including some medical services through the universal long-term care insurance system. Care managers (CMs) are the professionals responsible for introducing and managing these services. CMs play a vital role in care planning, management, family support, and service coordination for individuals requiring assistance [6-8]. In the field of home palliative care, in particular, CMs must possess a solid understanding of medical knowledge and the roles of medical professionals. However, the content of the care plan may vary depending on a CM’s work experience, work style, and previous background in the medical or nursing fields. In the field of rehabilitation, the Classification of Cancer Rehabilitation by Dietz [9] divides rehabilitation into four phases: preventive, restorative, maintenance, and palliative, indicating the role rehabilitation should play at all stages of the disease. Palliative rehabilitation, one of these phases, is essential for maintaining and improving quality of life (QOL) while respecting the wishes of terminally ill patients with cancer and their families [10]. Occupational therapy in palliative care also aims to promote independent living and maintain QOL for as long as possible [11].

In many countries, including Japan, there is a growing recognition of the importance of providing optimal palliative care to support patients with advanced illnesses and their caregivers who wish to remain at home [5]. Several palliative care initiatives for the community have been implemented in Japan as well [12,13], and improved communication and cooperation among regional healthcare professionals, along with increased confidence in the system’s ability to care for cancer patients at home, have been identified as key outcomes of these regional palliative care programs. However, despite these efforts, palliative rehabilitation in the home setting remains under-recognized and underutilized. It has been noted that CMs need to serve as coordinators of multidisciplinary teams, including medical and nursing care professionals. However, the main collaborators for CMs are often doctors and nurses, with low rates of collaboration with therapists [14]. As noted, collaboration with the CM is critical for implementing effective home-visit rehabilitation for terminally ill patients with cancer. Despite this necessity, there is a lack of studies investigating the background and challenges preventing progress in this area. Therefore, it is pertinent to clarify the current situation to address these issues effectively.

Therefore, this study aimed to clarify the current status of home-visit rehabilitation for terminally ill patients with cancer, focusing on CMs. By identifying associated factors, we aim to suggest guidelines for promoting the use of home-visit rehabilitation to maintain and improve the QOL of terminally ill patients with cancer in their home settings.

## Materials and methods

Survey participants

The survey targeted CMs operating in western Japan within the service areas of home care support clinics that offer home-visit rehabilitation services and promote home medical care.

Survey methodology

The purpose of the survey was explained in advance. Unmarked, self-administered survey forms were then distributed through the staff of home-visit rehabilitation facilities that agreed to participate (see Appendix for the survey (Appendix A - Japanese version, Appendix B - English version)). These forms were distributed either by mail or in person, and completed surveys were collected via the same methods. We did not send out reminders by mail.

Survey items

The survey included the following 11 questions: gender, age, years of work experience, basic job title, work style, office management system, home-visit rehabilitation experience, home-visit rehabilitation experience for terminally ill patients with cancer, perception and importance of home-visit rehabilitation for terminally ill patients with cancer, and reasons for not implementing home-visit rehabilitation for a terminally ill cancer patient.

The perception of home-visit rehabilitation for terminally ill patients with cancer was rated on a four-point scale from “not at all” (one point) to “very much” (four points). The importance of home-visit rehabilitation for terminally ill patients with cancer was rated on a four-point scale from “not at all” (one point) to "very much” (four points). In addition, respondents were asked to provide their reasons for not implementing home-visit rehabilitation for terminally ill patients with cancer.

The survey items were finalized after consultation with a co-researcher specializing in end-of-life and palliative care. A pretest involving six CMs working in the field was conducted to assess the clarity of the questions, ease of response, and content validity.

Statistical analysis

Initially, we calculated basic statistics for the participants' attributes and the scores for each survey question. A t-test was conducted, after confirming normality, to compare the differences in age and years of work experience between participants with and without experience in home-visit rehabilitation (including for terminally ill patients with cancer).

In addition, we conducted a chi-square test to determine whether there was an association between home-visit rehabilitation experience and office management system (single or establishment), working form (full-time or part-time), and basic qualifications (medical profession or care worker). Another chi-square test examined whether differences existed in the perception and importance of home-visit rehabilitation for terminally ill patients with cancer based on the presence or absence of home-visit rehabilitation experience for this patient group.

We used IBM SPSS Statistics for Windows, version 24.0 (released 2015, IBM Corp., Armonk, NY) for all statistical analyses. All p-values were two-tailed, with significance set at p < 0.05.

Ethical considerations

This study was approved by the Ethical Review Committee of Call Medical Clinic Fukuoka (approval number: YC 2013-01). The questionnaire was anonymous and accompanied by a letter of intent explaining the purpose of the study and privacy protection measures. Each questionnaire was mailed with a note emphasizing consideration for the respondents. Returning the completed questionnaire was considered as providing consent.

## Results

Response rate

Out of 520 questionnaires distributed across seven regions in three prefectures, 388 were returned, resulting in a response rate of 74.6%. After excluding incomplete responses, data from 357 respondents were considered valid.

Respondents’ characteristics

Table 1 presents the demographic characteristics of the respondents. Of the 357 respondents, 56 (15.7%) were men, and 301 (84.3%) were women. The average age was 47.8 ± 9.4 years, ranging from 27 to 70. Individuals in their 40s and 50s accounted for 65.5% of the sample. The mean number of years of work experience was 5.5 ± 3.8 years, with 121 (33.9%) having less than four years of experience.

**Table 1:** Demographic characteristics of the respondents

Item (mean ± SD)		N	%
Sex	Men	56	15.7
	Women	301	84.3
Age (47.8 ± 9.4)	20-29 years old	3	0.01
	30-39 years old	75	21
	40-49 years old	115	32.2
	50-59 years old	119	33.3
	60-69 years old	44	12.3
	Over 70 years old	1	0
Years of work experience	Less than one year	18	5.0
(5.5 ± 3.8)	～Less than four years	121	33.9
	～Less than seven years	88	24.6
	～Less than 10 years	60	16.8
	～Less than 13 years	56	15.7
	～Less than 14 years	14	3.9
Working form	Full-time employment	315	88.2
	Part-time work	42	11.8
Office management system	Single	95	26.6
	Establishment	262	73.4
Home-visit rehabilitation	Yes	299	83.8
experience	No	58	16.2
Home-visit rehabilitation	Yes	98	27.5
experience for terminally ill	No	259	72.5
patients with cancer			
Image of home-visit	I can't picture it at all.	10	2.8
rehabilitation for terminally	I can't imagine much.	121	33.9
ill patients with cancer	I can imagine a few.	187	52.4
	I can image very much.	39	10.9
Necessity for home-visit	I don't feel it at all.	5	1.4
rehabilitation for terminally	I don't feel much.	88	24.6
ill patients with cancer	I feel it a little.	200	56.0
	I feel it very much.	64	17.9

Regarding home-visit rehabilitation experience, 299 respondents (83.8%) answered affirmatively, while 58 (16.2%) indicated no experience. For home-visit rehabilitation specifically for terminally ill patients with cancer, 259 (72.5%) responded negatively, while 98 (27.5%) had experience in this area.

The distribution by occupation is presented in Table 2. In total, 92 respondents (23.3%) were in the medical profession, while 296 (74.9%) were in the nursing profession. Among them, 223 (56.5%) were caregivers, representing 56.5% of the total (7 non-respondents were excluded).

**Table 2:** Occupational distribution of the respondents

Basic qualifications	N	Classification	%
Nurse	54		
Practical nurse	10		
Public health nurse	6		
Dental hygienist	8		
Pharmacist	3		
Nutritionist	5		
Registered Dietitian	3		
Occupational therapist	2		
Physical therapist	1		
Total	92	Medical profession	23.3
Social worker	51		
Care worker	223		
Social welfare officer	8		
Helper level 2	5		
Helper level 1	2		
Livelihood support consultant	3		
Psycho-social worker	3		
Kindergarten teacher	1		
Total	296	Nursing profession	74.9

Factors associated with home-visit rehabilitation experience

The mean age of the respondents with experience in home-visit rehabilitation was 48.5 ± 9.0 years, compared to 44.1 ± 10.3 years for those without experience, indicating a significant difference between the two groups. Similarly, the mean years of work experience for respondents with home-visit rehabilitation experience were 5.9 ± 3.7 years, compared to 3.2 ± 3.5 years for those without experience, also showing a significant difference. In terms of working form, respondents with experience were highly represented across both full-time and part-time work, as well as full-time and concurrent work, with significant differences observed However, no significant relationships were found for office type and basic qualifications (Table 3).

**Table 3:** Factors associated with home-visit rehabilitation experience a: t-test, b: chi-square test

	Experienced	No experience	p^a^
Mean age (standard deviation)	48.5 (9.0)	44.1 (10.3)	0.004
Mean years of work experience (standard deviation)	5.9 (3.7)	3.2 (3.5)	＜0.001
	N (%)	N (%)	p^b^
Office management system			0.746
Single	81 (27.1)	14 (24.1)	
Establishment	218 (72.9)	44 (75.9)	
Working form			＜0.001
Full-time	272 (86.3)	43 (13.7)	
Part-time	27 (64.3)	15 (35.7)	
Basic qualifications			0.602
Medical profession	67 (82.7)	14 (17.3)	
Care worker	228 (85.1)	40 (14.9)	

Factors associated with home-visit rehabilitation experience for terminally ill patients with cancer

The mean age of respondents with experience in home-visit rehabilitation for terminally ill patients with cancer was 49.2 ± 8.8 years, while for those without experience, it was 47.2 ± 9.5 years, exhibiting no significant difference. However, the mean years of work experience for respondents with experience was 6.9 ± 3.7 years, compared to 4.9 ± 3.6 years for those without experience, indicating a significant difference. In addition, there was a significant association between home-visit rehabilitation experience for terminally ill patients with cancer and both the perception of and necessity for such services (Table 4).

**Table 4:** Factors associated with home-visit rehabilitation experience for terminally ill patients with cancer a: t-test, b: chi-square test

	Experienced	No experience	p^a^
Mean age (standard deviation)	49.2 (8.8)	47.2 (9.5)	0.064
Mean years of work experience (standard deviation)	6.9 (3.7)	4.9 (3.6)	＜0.001
	N (%)	N (%)	p^b^
Basic qualifications			0.087
Medical profession	28 (34.6)	53 (65.4)	
Care worker	66 (24.6)	202 (75.4)	
Perception			＜0.001
High	82 (36.3)	144 (63.7)	
Low	16 (12.2)	115 (87.8)	
Necessity			0.001
Yes	85 (32.2)	179 (67.8)	
No	13 (14.0)	80 (86.0)	

Reasons for not introducing home-visit rehabilitation for terminally ill patients with cancer (multiple answers allowed)

**Figure 1:**
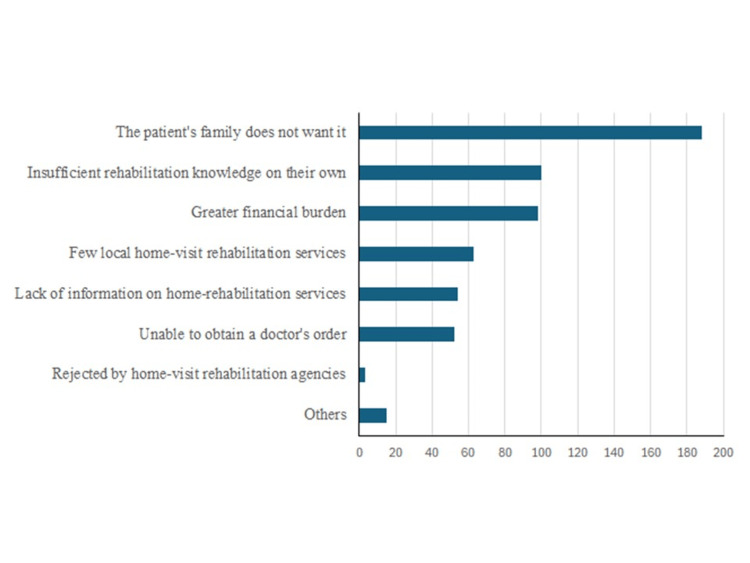
Reasons for not introducing home-visit rehabilitation for terminally ill cancer patients

The most frequently cited reason was “the patient's family does not want it,” followed by “lack of knowledge about rehabilitation” and “increased financial burden” (Figure 1).

## Discussion

Home-visit rehabilitation

Our study revealed that age, years of work experience, and working form were associated with home-visit rehabilitation experience among CMs. Older workers and those with more years of experience tended to have more opportunities for home-visit rehabilitation. This trend may be attributed to the diverse care plans that experienced CMs have encountered throughout their careers. In terms of working form, full-time workers had a higher percentage of home-visit rehabilitation experience compared to part-time workers. This could be due to full-time workers being involved in more cases as care support specialists, working longer hours, and having easier access to knowledge and information on local medical and nursing care, including home-visit rehabilitation.

Studies have reported that joint range of motion training, muscle strengthening, and walking are the top three expected components of home-visit rehabilitation according to CMs, with an emphasis on functional recovery training [15]. In a survey of users, prevention of functional deterioration and exercise guidance were also cited as reasons for initiating home-visit rehabilitation programs [16]. However, in addition to these functional approaches, home-visit rehabilitation encompasses physical therapy to manage movement, occupational therapy to facilitate environmental engagement and independent activities, and speech-language therapy to improve eating, swallowing, and communication. Conducting lifestyle function assessments to address activity limitations and participation restrictions in real-life daily situations is also integral to home-visit rehabilitation.

Another crucial role of CMs is to reduce the burden of caregiving by offering referrals and guidance to family members and caregivers on how to provide assistance. However, inadequate service infrastructure presents a challenge, characterized by a shortage of human resources in both home-visit rehabilitation facilities and therapists, as well as the current dependence on the discretion of CMs [7]. This study highlights the impact of CMs’ knowledge and experience on the implementation of home-visit rehabilitation. To promote home-visit rehabilitation effectively, it is essential for each therapist to have a clearly defined and unique role, and for professionals to articulate the content and goals of home-visit rehabilitation in a manner that CMs and other home support staff can easily understand. This collaborative approach is crucial for the successful promotion of home-visit rehabilitation.

Home-visit rehabilitation for terminally ill patients with cancer

Despite a high perceived value and necessity for home-visit rehabilitation for terminally ill patients with cancer, the survey revealed that more than 70% of respondents had not implemented such services. The main reasons cited were "the patient's family does not want it," "lack of knowledge about rehabilitation," and "increased financial burden."

Regarding “the patient's family does not want it,” many families may associate rehabilitation strongly with functional training, making it difficult to envision what it entails at home. This challenge may also arise when “one's own knowledge of rehabilitation is insufficient,” making it hard for CMs to explain its role to patients and families. This suggests a lack of opportunities to establish a shared understanding of the content and role of home-visit rehabilitation services for terminally ill patients with cancer. Therapists need to actively engage with CMs to address this gap.

The issue of "financial burden" is not limited to terminally ill patients with cancer but extends to those in the maintenance phase as well [15]. CMs must tailor care plans within the patient’s financial means. This reflects the CMs’ empathetic approach, considering the financial strain on patients and families, particularly during the terminal stage of cancer when both nursing and medical costs increase. These findings underscore the importance of clearly defining the role of rehabilitation and emphasizing its necessity.

No association was found between basic qualifications and home-visit rehabilitation experience for terminally ill patients with cancer, consistent with the results of a previous study [14]. However, this study is the first to identify an association with years of work experience. In addition, individuals with experience in home-visit rehabilitation tended to have a more positive perception and a greater sense of its necessity compared to those without such experience. These findings suggest that understanding of home-visit rehabilitation for terminally ill patients with cancer is gradually deepening through the practical experiences and involvement of CMs.

This study was conducted in a limited area covering three prefectures and seven regions, which restricts the generalizability of the findings. To obtain broader insights, a nationwide survey would be necessary. In addition, the reasons why terminally ill patients with cancer and their families may refuse home-visit rehabilitation have not been examined in this study. Future research should expand the participant pool and identify factors that either promote or hinder the adoption of home-visit rehabilitation for terminally ill patients with cancer. Despite these limitations, this study has provided valuable insights into the current challenges surrounding the implementation of home-visit rehabilitation for terminally ill patients with cancer in Japan. It is hoped that these findings will contribute to promoting home-visit rehabilitation to help maintain and improve the quality of life for terminally ill patients with cancer in the future.

## Conclusions

This study examined the factors associated with home-visit rehabilitation experiences among CMs. The results revealed that age, years of work experience, and work type were related to home-visit rehabilitation experience. A significant association was identified between home-visit rehabilitation experience for terminally ill patients with cancer and years of work experience, as well as the perception and necessity for home-visit rehabilitation. These results suggest that to promote the use of home-visit rehabilitation for terminally ill patients with cancer, it is necessary to encourage CMs without experience in introducing this service to deepen their shared understanding of its benefits.

## Figures and Tables

**Figure 2:**
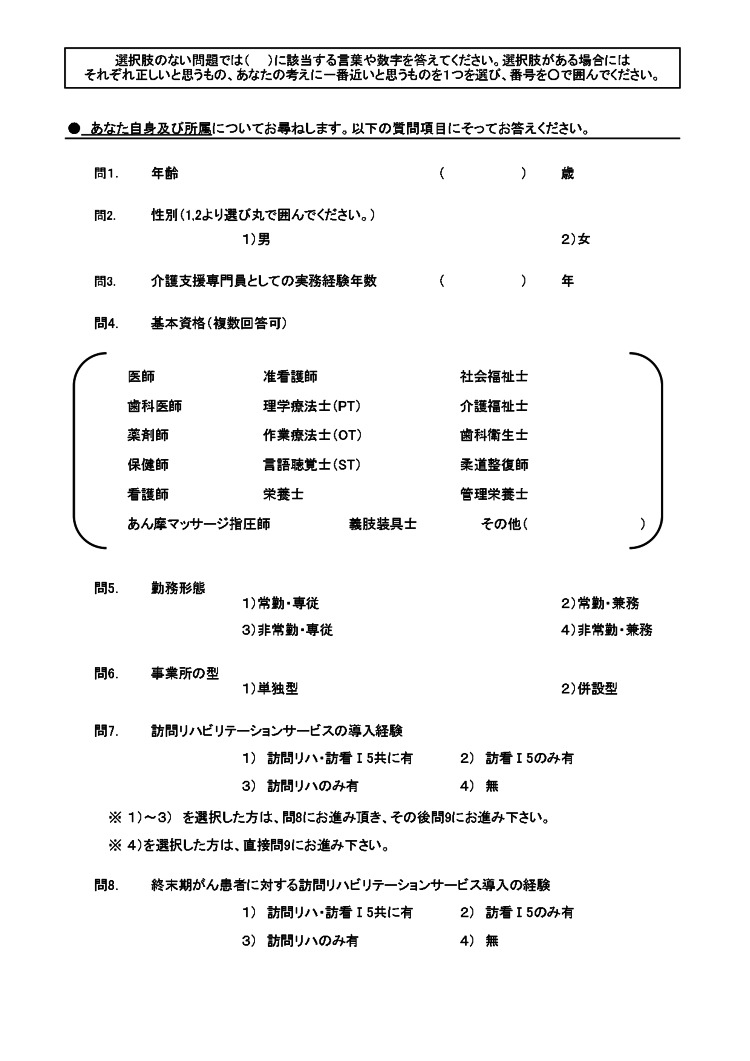
Survey form - Japanese version (page 1)

**Figure 3:**
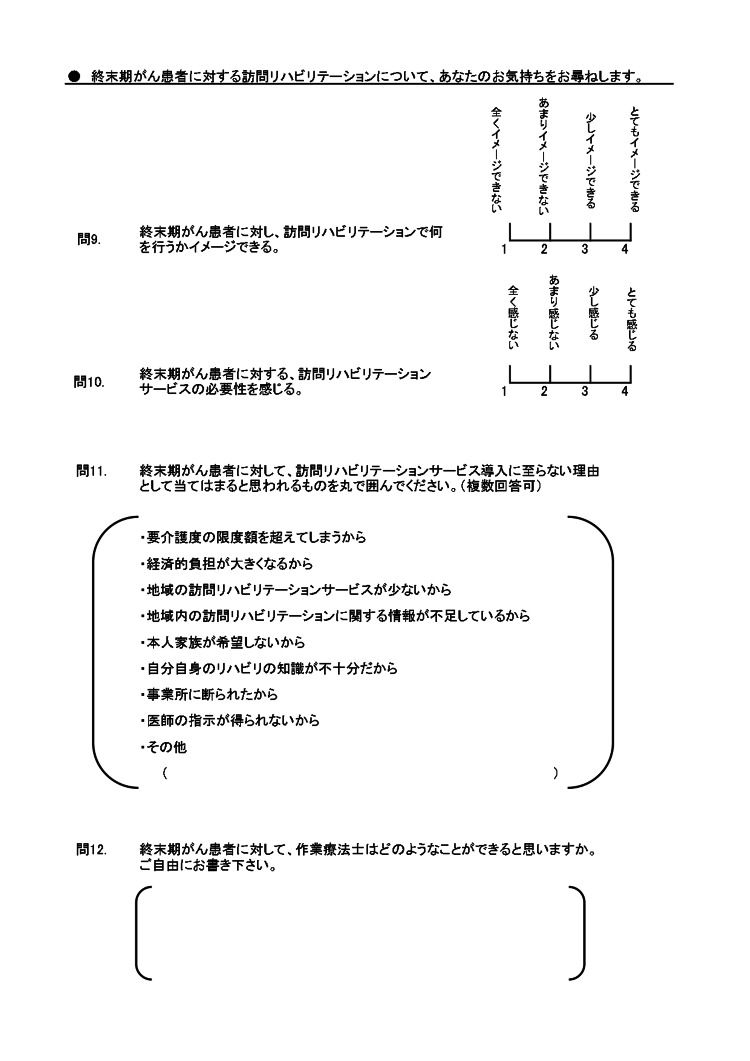
Survey form - Japanese version (page 2)

**Figure 4:**
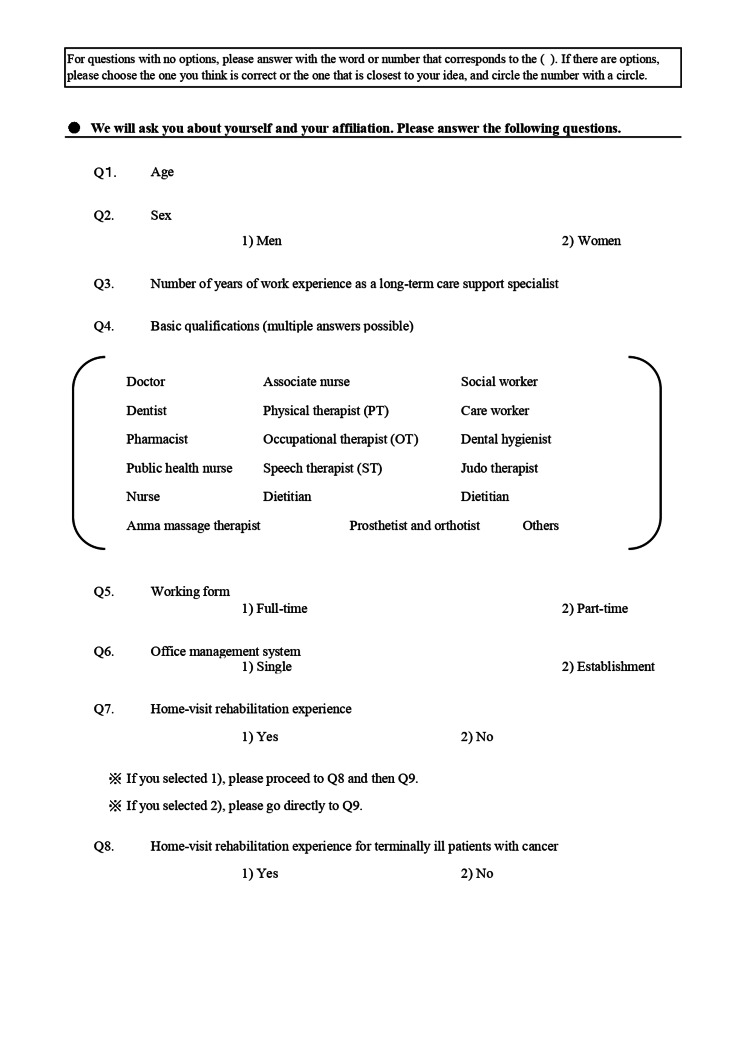
Survey form - English version (page 1)

**Figure 5:**
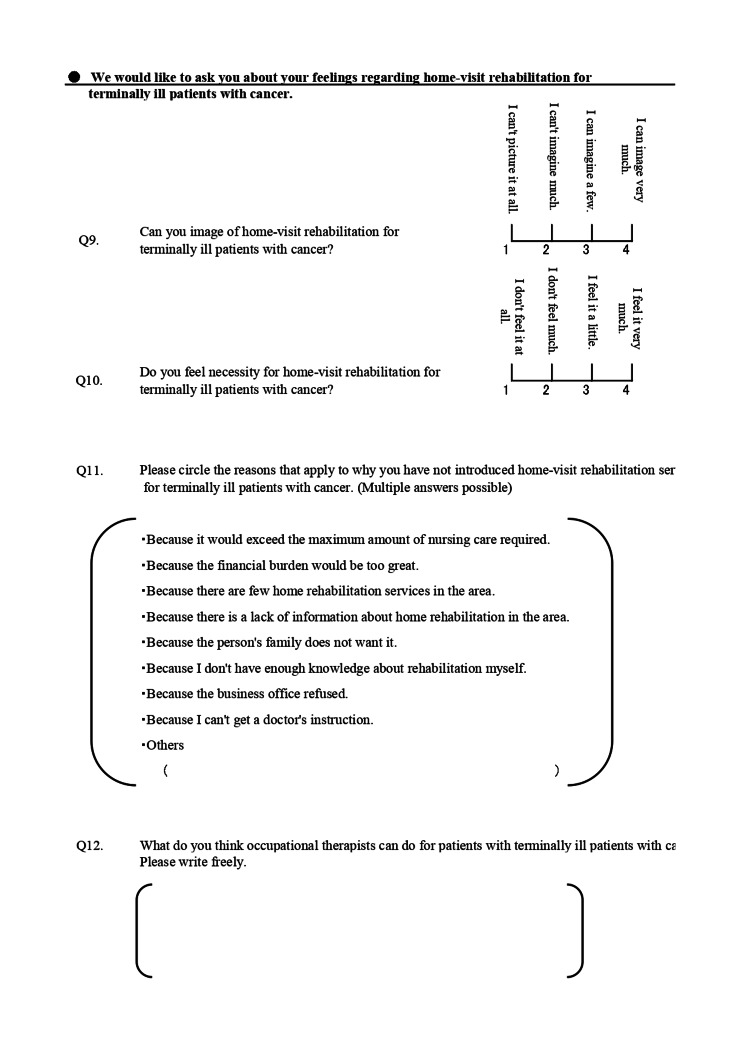
Survey form - English version (page 2)

## Appendices

Appendix A

Appendix B

## References

[1] (2024). White paper on health, labour and welfare - a social model to overcome population aging (in Japanese). https://www.mhlw.go.jp/content/000351675.pdf.

[2] (2024). Ministry of Health, Labour and Welfare (2017). National Institute of Population and Social Security Research: population projections for Japan: 2016-2065 (with long-range population projections 2066-2115) (in Japanese). https://www.ipss.go.jp/pp-zenkoku/j/zenkoku2017/pp29_ReportALL.pdf.

[3] (2024). Ministry of Health, Labour and Welfare (2009). Report on the fiscal year 2009 survey on attitudes toward medical care in the last stage of life (in Japanese). https://www.mhlw.go.jp/toukei/list/dl/saisyuiryo_a_h29.pdf.

[4] Gomes B, Calanzani N, Curiale V, McCrone P, Higginson IJ (2013). Effectiveness and cost-effectiveness of home palliative care services for adults with advanced illness and their caregivers. Cochrane Database Syst Rev.

[5] World Health Organization (2011 (2024). Palliative care for older people: better practices. https://iris.who.int/bitstream/handle/10665/326378/9789289002240-eng.pdf?sequence=1.

[6] Susa K, Sakaya S, Murata H (2009). Care manager’s consideration of terminal care system issue for home elderly. Bulletin of Dokkyo Medical University School of Nursing.

[7] Takahashi Y (2009). Palliative home care management by a care manager [Article in Japanese]. Palliative Care.

[8] van der Plas AG, Vissers KC, Francke AL, Donker GA, Jansen WJ, Deliens L, Onwuteaka-Philipsen BD (2015). Involvement of a case manager in palliative care reduces hospitalisations at the end of life in cancer patients; A mortality follow-back study in primary care. PLoS One.

[9] Dietz JH (1981). Rehabilitation oncology. https://www.ncbi.nlm.nih.gov/nlmcatalog/8009361.

[10] Yomiya K (2008). Palliative care [Article in Japanese]. Sogo Rihabiriteshon.

[11] Cooper J (2006). Occupational therapy in oncology and palliative care (2nd ed). https://www.wiley.com/en-es/Occupational+Therapy+in+Oncology+and+Palliative+Care%2C+2nd+Edition-p-9780470019627.

[12] Imura C, Morita T, Kato M (2014). How and why did a regional palliative care program lead to changes in a region? A qualitative analysis of the Japan OPTIM study. J Pain Symptom Manage.

[13] Morita T, Miyashita M, Yamagishi A (2012). A region-based palliative care intervention trial using the mixed-method approach: Japan OPTIM study. BMC Palliat Care.

[14] Ohta R, Ryu Y, Katsube T (2019). Care managers in rural Japan: challenges to interprofessional collaboration. Home Health Care Serv Q.

[15] Horino K (2007). Promotion of the use of home-visit rehabilitation [Article in Japanese]. J Phys Ther Res.

[16] Fujita Y, Tanaka Y, Takeda H (2011). The realities and problems of long-term care insurance use for terminal cancer patients. Jap J Care Manag.

